# Vermicompost Supplementation Improves the Stability of Bioactive Anthocyanin and Phenolic Compounds in *Clinacanthus nutans* Lindau

**DOI:** 10.3390/molecules23061345

**Published:** 2018-06-04

**Authors:** Zuhaili Yusof, Sujatha Ramasamy, Noor Zalina Mahmood, Jamilah Syafawati Yaacob

**Affiliations:** 1 Institute of Biological Sciences, Faculty of Science, University of Malaya, Kuala Lumpur 50603, Malaysia; zuhailiyusof@yahoo.com (Z.Y.); sujatha@um.edu.my (S.R.); alin@um.edu.my (N.Z.M.); 2 Centre for Research in Biotechnology for Agriculture (CEBAR), Institute of Biological Sciences, Faculty of Science, University of Malaya, Kuala Lumpur 50603, Malaysia

**Keywords:** total phenolic content, anthocyanin, flavonoid, antioxidant, extract storage, vermicompost, *Clinacanthus nutans*

## Abstract

This project studied the effect of vermicompost application on the composition of bioactive anthocyanin and phenolic compounds, and the antioxidant activity of *Clinacanthus nutans*. The correlation between the bioactive constituents and antioxidant capacity was also evaluated. In this project, a field study was conducted using a randomized complete block design (RCBD) with four treatment groups, including control plants (CC), plants supplied with chemical fertilizer (CF), plants supplied with vermicompost (VC), and plants supplied with mixed fertilizer (MF). The leaves of *C. nutans* from all treatment groups were harvested, subjected to solvent extraction, and used for quantification of total anthocyanin content (TAC), total phenolic content (TPC), and total flavonoid content (TFC). The initial antioxidant activity of the extracts was evaluated using 2,2-Diphenyl-1-picrylhydrazyl (DPPH) and 2,2′-azinobis(3-ethylbenzothiazoline-6-sulfonic acid) (ABTS) assays, as well as after two and four weeks of storage at −20 °C and 4 °C. Data analysis showed that CC plants contained the highest TAC (2180.14 ± 338.43 µg/g dry weight) and TFC (276.25 ± 3.09 mg QE/g dry weight). On the other hand, CF plants showed the highest TPC (181.53 ± 35.58 mg GAE/g dry weight). Moreover, we found that CC plants had the highest antioxidant potential against DPPH radicals whereas MF plants showed the lowest antioxidant potential. After four weeks of extract storage at −20 °C and 4 °C, the TPC, TFC, TAC, and antioxidant potential of the extracts decreased. Extracts from VC showed the lowest percentage of total phenolic and total flavonoid loss after extract storage at −20 °C and 4 °C compared with other plant extracts. At this juncture, it could be deduced that the application of vermicompost had little effect on the expression of phenolics, flavonoids, or anthocyanin in *C. nutans*. However, the extract from plants treated with vermicompost (VC and MF) showed better stability compared with CC and CF after extract storage at different temperatures.

## 1. Introduction

*Clinacanthus nutans* Lindau is an important medicinal plant from the family Acanthaceae. A tall, erect herbaceous perennial shrub, it grows up to one meter in height and is distributed throughout tropical regions, including Southeast Asia and China. The plant is locally known in Malaysia as “Belalai Gajah” due to the slightly curved stem supporting the leaves that resembles the curve of an elephant’s trunk [1]. In Malaysia and Thailand, the leaves of *C. nutans* are used as a remedy against venomous snake bites, scorpion, and insect stings [2,3], resulting in the vernacular name, Sabah snake grass. *C. nutans* is classified as one of Malaysia’s high-value herbal products in the Entry Point Project 1 (EPP1) in the agriculture sector, which is one of the identified National Key Economic Areas (NKEAs). *C. nutans* has been reported to contain various phytochemicals with biological activities including antioxidant [4,5], antimicrobial [6], anti-inflammatory [5,7], antivenom [2], and anticancer activities [3,8]. This plant has also been widely used for the treatment of various diseases such as cancer, herpes simplex virus (HSV), varicella-zoster virus (VZV) lesions, skin rashes, and kidney problems [1,3].

Researchers are increasingly embracing green technology and the usage of sustainable and environmentally friendly approaches to enhance the production of valuable secondary metabolites in plants. Plants produce essential secondary metabolites for their adaptation and defense against environmental stressors. For example, water-soluble flavonoids play an important role in protecting plant’s cellular processes against ultraviolet-B (UVB) radiation [9]. Flavonoids are used in various industries, such as in the food industry as a food additive. In addition, flavonoids are used in the production of pharmaceutical products [10] and as a source of environmentally friendly colorants in the textile industry [11]. Among the pigments synthesized via the flavonoid pathway, anthocyanins are one of the most important. Anthocyanins can appear red, blue, or purple and have the potential to be a natural food colorant [12]. Anthocyanins have been reported to help in improving cardiovascular disease and cancer [13,14]. The vast benefits of anthocyanins have rendered them a healthier alternative to synthetic dyes and food colorants in the growing food and beverage industry, as the color property is seen as an important factor in influencing consumer acceptance and to reflect the quality of the product [15,16]. Moreover, advances in the use of natural dyes as functional colorants have been made in various sectors, such as in dye-sensitized solar cells, development of biomedical sensors, and optical data storage [17,18,19,20]. The applications of plant-derived natural colorants or dyes for green technology development appears to be limitless as demands for sustainable production of these valuable pigments continue to increase. Thus, “green” practices in the synthesis of these valuable pigments, such as through organic production, are key to increase consumer acceptance, especially for its application in the food industry.

In this study, the effects of organic growth supplements, including vermicompost in accumulation of bioactive phenolic, anthocyanin, and flavonoid compounds in *C. nutans*, as well as their antioxidant potential were evaluated. Vermicompost is an organic materials broken down by the interactions between microorganisms and earthworms in a mesophilic process to produce fully stabilized and organic soil amendments with low carbon to nitrogen (C:N) ratios [21]. Vermicompost is rich in NPK (nitrogen 2–3%, phosphorus 1.55–2.25%, and potassium 1.85–2.25%), micronutrients, and beneficial soil microbes, so it is garnering attention as a greener replacement for chemical fertilizers to maintain and further improve soil quality. The application of vermicompost as an alternative to chemical fertilizer not only produces healthier plants, but it also increases plant resistance toward pests and diseases. Moreover, vermicomposting is a quicker and more cost-effective technique for composting as it helps in diverting organic waste from landfills. Vermicompost has been reported to significantly stimulate the growth of a wide range of plant species including several medicinal plants [22], horticultural crops [23,24,25,26], fruit crops [27,28], ornamentals [29,30], and forestry species [31,32,33]. However, few publications were found on the effect of vermicompost supplementation on the availability of phytoconstituents and bioactivity of *C. nutans*. Previous works conducted on the application of vermicompost only focused on the effects of its supplementation on plant growth. The outcomes of this study provide additional knowledge and understanding about the effects of vermicompost supplementation on the availability of bioactive phenolic and anthocyanin compounds in *C. nutans*. Thus, this study adds to the knowledge that can be used for ensuring sustainable production of these bioactive pigments from natural sources, while reducing the impact on the environment.

## 2. Materials and Methods

### 2.1. Sample Preparation

In this project, a field study using a randomized complete block design (RCBD) with four treatment groups was conducted at Glami Lemi Biotechnology Research Centre (PPBGL), Jelebu, Malaysia. The treatment groups included control plants (CC), plants supplied with 10 t/ha of NPK fertilizer (CF), plants supplied with vermicompost at 15 t/ha (VC), and plants supplied with mixed fertilizer (containing both 15 t/ha of vermicompost and 5 t/ha of NPK fertilizer; MF). All treatments were applied on the plots as a soil conditioner two weeks before the planting process. The leaves from three *C. nutans* plants of each of the three different blocks per treatment were harvested in February 2017, resulting in a total of nine samples for each treatment. *C. nutans* was identified by comparing the plant with a herbarium specimen and a voucher specimen (KLU 49509) was deposited at the Herbarium of Rimba Ilmu, Institute of Biological Sciences, University of Malaya, Kuala Lumpur, Malaysia. After harvesting the leaves, the methanolic extract of *C. nutans* was prepared, in which the fresh leaves were freeze-dried and subjected to solvent extraction using methanol. Briefly, 3 g of freeze-dried leaves were soaked in 90 mL 70% methanol and ground using a mortar and pestle. Then, the sample mixture was incubated in solvent at 4 °C for 24 h followed by filtration using filter paper. The extraction process was repeated using the residue obtained from the filtration. The filtrates were pooled and evaporated to dryness using a rotary evaporator at 45 °C to yield the methanolic extracts. The concentrated extract was adjusted to a concentration of 20 mg/mL using 70% methanol before it was stored at either 4 °C or −20 °C until further analysis.

### 2.2. Phytochemical Screening of Bioactive Compounds in C. nutans

The presence of various phytochemicals such as alkaloid, tannin, phenol, flavonoid, and saponin in the samples was evaluated based on standard methods [34].

#### 2.2.1. Measurement of Total Anthocyanin Content

The total anthocyanin content was determined using the pH differential method [35]. The methanolic extracts of *C. nutans* were diluted separately with two types of buffer: potassium chloride (0.025 M) at pH 1.0 and sodium acetate (0.4 M) at pH 4.5 using the ratio 1:4 (1 part test portion and 4 parts buffer). The absorbance was measured at wavelengths of 520 nm and 700 nm using UV-200-RS spectrophotometer (MRC Ltd., Holon, Israel). The concentration of anthocyanin pigment was measured using the following formula:

$$\mathrm{Anthocyanin}\;\mathrm{pigment}\;\mathrm{content}\;\left(\mathrm{mg}/L\right)\;=\;\frac{\left(A\times \mathrm{MW}\times \mathrm{DF}\times 1000\right)}{\left(\varepsilon \times 1\right)}$$

$$\mathrm{where}\;A=\left({\mathrm{Abs}}_{520}-{\mathrm{Abs}}_{700}\right)\mathrm{pH}1.0-\left({\mathrm{Abs}}_{520}-{\mathrm{Abs}}_{700}\right)\mathrm{pH}4.5$$

MW(cyanidin−3−glucoside)=449.2 g/mol

DF=dilution factor

ε=26,900

#### 2.2.2. Measurement of Total Phenolic Content

Folin-Ciocalteu’s method was used to quantify the total phenolic content of *C. nutans* according to the method described by Sun, etal. [36] with minor changes. Briefly, Folin-Ciocalteu reagent was diluted 10-fold with deionised water. A total of 0.1 mL of *C. nutans* methanolic extract was mixed with 0.75 mL of diluted Folin-Ciocalteu reagent and incubated for 10 min at room temperature. Then, 0.75 mL of 2% sodium carbonate (Na_2_CO_3_) solution was added. The mixture was allowed to stand in the dark for 45 min before measuring the absorbance at 765 nm using a UV-200-RS spectrophotometer (MRC Ltd., Holon, Israel) against a blank, containing the solvent (70% methanol). The TPC of the samples was determined from a calibration curve prepared with a series of gallic acid standards (0.01, 0.02, 0.03, 0.04, 0.05, and 0.06 mg/mL). Results were expressed as mg of gallic acid equivalents/g dry weight (mg GAE/g DW) of extract.

#### 2.2.3. Measurement of Total Flavonoid Content

The aluminum chloride colorimetric method [37] was used to quantify the total flavonoid content in the methanolic extracts of *C. nutans.* First, 0.5 mL of each extract was mixed with 1.5 mL of 70% methanol, 0.10 mL of 10% aluminum chloride, AlCl_3_ (AlCl_3_·6H_2_O), 0.10 mL of sodium acetate (NaC_2_H_3_O_2_·3H_2_O) (1 M), and 2.80 mL of distilled water. Then, the absorbance was measured at 415 nm using a UV-200-RS spectrophotometer (MRC Ltd., Holon, Israel) after 40 min of incubation. The flavonoid concentrations were calculated by preparing a calibration curve using quercetin as the standard (0.15–0.4 mg/mL). The flavonoid concentration was expressed as quercetin equivalents in mg per gram of dry weight (mg/g DW) of extract. All assays were performed in triplicate.

### 2.3. DPPH (2,2-Diphenyl-1-picrylhydrazyl) Radical Scavenging Activity Assay

DPPH free radical scavenging activity of the *C. nutans* methanolic extracts was analyzed following standard procedure [38]. First, 50 µL of extract at six different concentrations (0.5, 1.0, 1.5, 2.0, 2.5, and 3.0 mg/mL) were added to 150 µL of DPPH solution (60 mM) in each well of a 96-well plate. The mixture was then incubated at room temperature for 30 min. Then, 50 µL of methanol was added to the DPPH solution as a blank. At the end of the incubation period, a Multiskan Go plate reader (Thermo Scientific, Waltham, MA, USA) was used to measure the absorbance at 515 nm. All the extracts were assayed in triplicate. The obtained data were then used to determine the concentration of the sample required to scavenge 50% of the DPPH free radicals (IC_50_). The percentage of inhibition was plotted against the concentration and the IC_50_ was obtained from the fitted linear curve. A lower IC_50_ denotes a more potent antioxidant.

### 2.4. ABTS (2,2′-Azinobis(3-ethylbenzothiazoline-6-sulfonic acid)) Radical Scavenging Activity Assay

The colorimetric method described by Shao et al. [39] was used to perform the ABTS scavenging activity assay, with slight modifications. First, the preparation of the ABTS radical cation was performed by mixing 10 mL of 2.6 mM K_2_S_2_O_4_ solution with 10 mL of 7.4 mM ABTS solution. Then, the mixture was stored for 12 h at room temperature in a dark room before further use. After that, double distilled water (ddH_2_O) was used to dilute the mixture and it was adjusted to produce an absorbance reading of 0.70 ± 0.2 at 734 nm. A 20 µL of sample at six different concentrations (0.5, 1, 1.5, 2.0, 2.5, and 3.0 mg/mL) was then added to 200 µL ABTS solution and the mixture was incubated at room temperature for 30 min. The absorbance reading was measured at 734 nm using a Multiskan Go plate reader (Thermo Scientific, Waltham, MA, USA) and the assay was performed in triplicate. The percentage of inhibition was calculated according to the following formula:

$$\%\;\mathrm{inhibition}=\;\frac{\left({\mathrm{Abs}}_{\mathrm{blank}\;}-{\;\mathrm{Abs}}_{\mathrm{sample}}\right)}{\left({\mathrm{Abs}}_{\mathrm{blank}}\right)}\;\times \;100$$

The antioxidant capacity of the test extracts is expressed as IC_50_, which is the concentration necessary for a 50% reduction in ABTS radicals.

### 2.5. Extract Stability after Storage at Different Temperatures

In this study, the effect of the growth supplements on the stability of the methanolic extracts following extract storage at different temperatures was analyzed. For this, the extracts were stored at 4 °C or −20 °C for 2 and 4 weeks and then used in the determination of phenolic, anthocyanin and flavonoid contents. The effect of extract storage on antioxidant properties of the extracts was also determined.

### 2.6. Statistical Analysis

The data obtained in this study were subjected to statistical analysis using analysis of variance (ANOVA) and the mean values were compared using Duncan’s Multiple Range Test (DMRT) in SPSS software version 24. Pearson correlation analysis was also conducted to determine the relationship between the antioxidant potential with the amount of bioactive phenolic, anthocyanin, and flavonoid present in the extracts.

## 3. Results and Discussion

### 3.1. Phytochemical Screening

Qualitative screening of *C. nutans* methanolic extract was performed using standard protocols [34]. Results of the present study revealed the presence of phenols, flavonoid, and saponin in the methanolic extracts of *C. nutans* leaves (Table 1) but were negative for alkaloid and tannin (Table 1). These results are in agreement with previous studies [25,40]. However, the methanolic extract of *C. nutans* has also been reported to contain steroids and triterpenes [40].

### 3.2. Determination of Pigments Content (Total Anthocyanin, Phenolic and Flavonoid)

The total anthocyanin (TAC), total phenolic (TPC), and total flavonoid (TFC) contents in the methanolic extracts of *C. nutans* were measured. The TPC was expressed as mg gallic acid (GAE) per g dry weight of the sample, whereas the TFC was expressed as mg quercetin (QE) per g dry weight of the sample. Based on Table 2, the highest TAC was obtained in CC plants (2180.14 ± 338.43 μg/g dry weight), followed by CF, VC, and MF (Table 2). However, data analysis revealed that the differences observed among the TAC of all samples were not statistically significant. Conversely, significantly higher TFC was found in the methanolic extracts of CC (276.25 ± 3.09 mg QE/g dry weight) and CF (256.66 ± 45.43 mg QE/g dry weight) plants, compared with VC and MF. CF plant extract was also observed to contain the highest TPC (181.53 ± 35.58 mg GAE/g dry weight), followed by CC, VC, and MF (Table 2).

### 3.3. Antioxidant Potential of C. Nutans Methanolic Extracts against DPPH and ABTS Radicals

The DPPH assay is typically based on the scavenging of free radicals and converting them to colorless products. When the methanolic extract of *C. nutans* reacts with DPPH solution, the free radicals are reduced by hydrogen donation to produce the reduced form of 1,1-diphenyl-2-picryl hydrazine (non-radical), indicated by the color change from violet to pale yellow [41]. The ABTS (2,2′-azinobis(3-ethylbenzothiazoline-6-sulfonic acid)) assay is a decolourization assay in which the stable radical is generated directly through the reaction of ABTS with potassium persulfate, which resulted in the production of blue or green ABTS chromophore, prior to reaction with the antioxidants. The free radical scavenging activity of the extracts against DPPH and ABTS is expressed as IC_50_ (mg per mL of extract), which is the concentration of antioxidant necessary to decrease the initial DPPH and ABTS concentration by 50%. The antioxidant activities of the extracts against both DPPH and ABTS radicals are displayed in Table 3. Data analysis revealed that the methanolic extract from control plants (CC) exhibited the highest free radical scavenging activity against DPPH and ABTS radicals, with an IC_50_ of 1.18 ± 0.05 mg/mL and 0.98 ± 0.05 mg/mL, respectively (Table 3). The antioxidant activity (denoted by IC_50_) of the methanolic extracts against both DPPH and ABTS radicals, in decreasing order, is CC, CF, VC, and MF. 

Based on the results of the present study, the antioxidant capacity of the methanolic extracts of *C. nutans* evaluated by the ABTS method was higher than that evaluated with the DPPH method. The DPPH assay underestimated the antioxidant capacity of the methanolic extracts of *C. nutans* by 18.44–33.53% compared to the ABTS assay. This observation is in agreement with a previous study by Almeida et al. [42] that showed that a higher antioxidant capacity was obtained for fresh Brazilian exotic fruits when evaluated by ABTS assay compared with evaluation using the DPPH method. This observation can be attributed to several factors, such as the wavelengths used during the spectrophotometric measurement in both assays, where 515 nm was used in DPPH assays and 734 nm was used in ABTS assays. Arnao [43] reported that the underestimation by DPPH assays could be due to the pigments contained in the colored extracts (such as carotenoids and anthocyanins) having overlapping absorbance spectra in the visible region with that of DPPH at 515 nm, thus interfering with the absorbance readings.

This underestimate could be due to the structural conformation of the antioxidants that influenced the reaction mechanism of free radical scavengers and DPPH. Larger molecules that have less access to the radical site have a lower antioxidant activity for a particular test compared with smaller molecules [44]. Otherwise, the underestimation could be due to the reactions of certain phenols such as eugenol and its derivatives that are reversible when reacted with DPPH, resulting in low readings of the antioxidant capacity [45]. However, DPPH assays also have an advantage over ABTS assays, as the DPPH free radicals can be directly acquired without preparation so they are ready to dissolve, whereas ABTS radical cations must be produced through enzymatic (peroxidase and myoglobin) or chemical (manganese dioxide and potassium persulfate) reactions [43]. Nevertheless, both the ABTS and DPPH assays have been the most popular spectrophotometric methods for determination of antioxidant capacity of foods and chemical compounds [46].

### 3.4. Effect of C. Nutans Extracts Storage (Duration and Temperature) on Stability of Pigments and Antioxidant Activity

**Figure 1:**
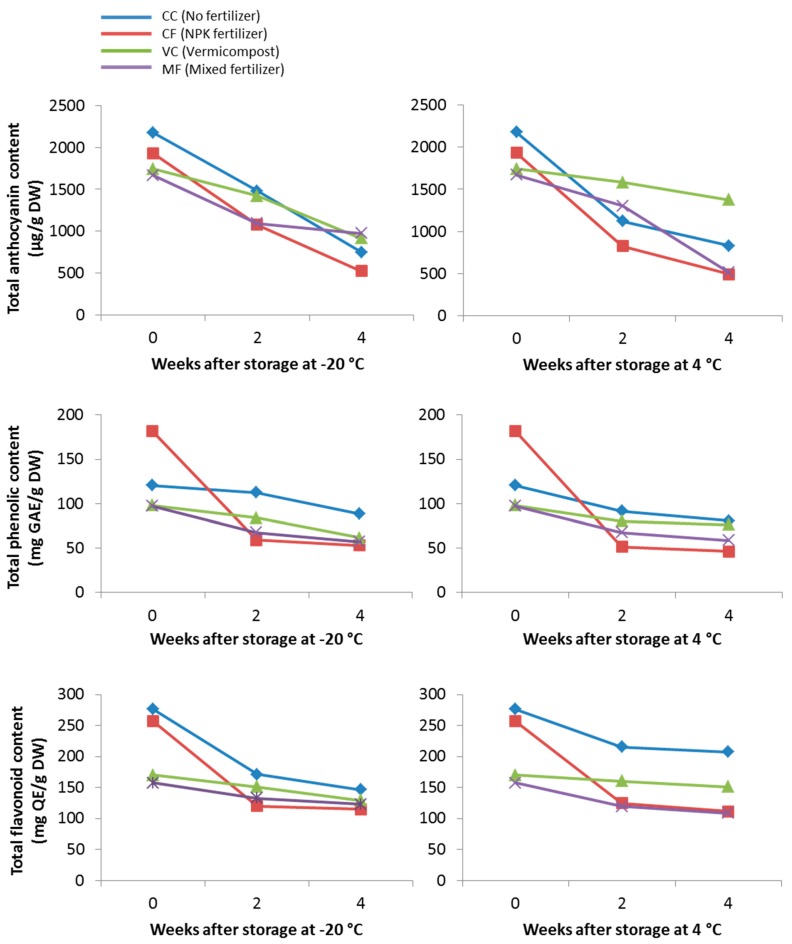
Effect of extract storage at −20 °C and 4 °C on total anthocyanin (TAC), total phenolic (TPC), and total flavonoid (TFC) contents in the methanolic extracts of *C. nutans* supplemented with different fertilizers.

In this study, the TPC, TAC, and TFC of the methanolic extracts after two and four weeks of storage at −20 °C and 4 °C were also evaluated. As shown in Figure 1, the TAC of all plant extracts decreased after four weeks of storage at −20 °C and 4 °C. More than 50% of the total anthocyanin loss for CC and CF plant extracts occurred after four weeks of storage at −20 °C and 4 °C compared to VC and MF. VC plant extract showed the lowest percentage of total anthocyanin loss (21.0%) after four weeks of storage at 4 °C, compared to other extracts that exhibited a TAC loss of more than 50%.

Data analysis showed that TPC of the extracts decreased gradually with extract storage (Figure 1). After 4 weeks of storage at −20 °C, the TPC was observed to decrease by 26.62%, 70.75%, 37.04% and 41.19% for CC, CF, VC, and MF extracts, respectively. Plants supplemented with vermicompost (VC) showed the least percentage of TPC loss after extract storage at 4 °C, compared to other plant extracts (Figure 1). It was also observed that plants supplemented with only NPK fertilizer (CF) showed the highest TPC loss after extract storage at both −20 °C and 4 °C (Figure 1). Similar results were observed for TFC, where CF plant extracts were found to exhibit the highest TFC loss after extract storage (Figure 1). Interestingly, data analysis again revealed that plants supplemented with vermicompost (VC) exhibited the lowest percentage of TFC loss, with a decrease of 23.96% and 11.30% after 4 weeks of storage at −20 °C and 4 °C, respectively.

**Figure 2:**
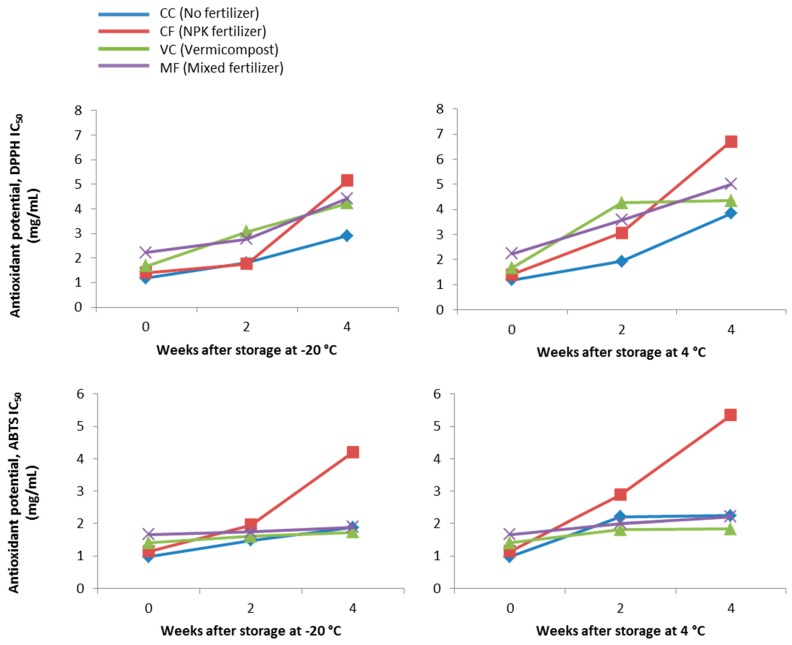
Effect of extract storage at −20 °C and 4 °C on antioxidant potential of the methanolic extracts of *Clinacanthus nutans* supplemented with different fertilizers.

Furthermore, the antioxidant potential of the plant extracts following extract storage at −20 °C and 4 °C were also monitored. Data analysis revealed that after 4 weeks of storage, the antioxidant potential of the extracts was significantly reduced, as denoted by the increase in IC_50_ values. As observed in Figure 2, plant extracts from CF showed the highest loss of antioxidant potential against DPPH and ABTS radicals, when stored at both −20 °C and 4 °C. It was also evident that supplementation with vermicompost (VC and MF) improved the stability of antioxidants present in the extracts (Figure 2). Based on correlation analysis (Table 4), the antioxidant potential exhibited by the plant extracts could be due to their pigments content (anthocyanin, phenolic and flavonoid), as shown by the negative correlations observed. The significant negative correlations indicate that the DPPH and ABTS IC_50_ values decreased with increasing anthocyanin, phenolic and flavonoid content. However, these correlations were only moderate, except for TAC, which was shown to be strongly correlated with DPPH IC_50_.

Based on the results obtained, vermicompost does not exert any significant effect on the expression of bioactive compounds, as evident by the lower values (TAC, TPC, and TFC) obtained for all vermicompost-treated plants. This finding is in agreement with a previous study conducted on lettuce (*Lactuca sativa* L.), which showed that vermicompost fertilization significantly increased the crop yield, but reduced the levels of phenolic compounds and antioxidant activity [47]. Similarly, pak choi (*Brassica rapa* cv Bonsai, Chinensis group) and chincuya (*Annona purpurea* Moc and Sesse ex Dunal) plants grown with vermicompost were also reported to contain lower amounts of phenolic compounds [48,49]. These observations indicate that some factors may be present in vermicompost that negatively interferes with the synthesis of phenolic compounds through the phenylpropanoid pathway [47]. The accumulation of these various secondary metabolites has been shown to be affected by the interactions between plant genotype (species and variety within species) and environmental factors, including cultivation technique, season, abiotic and biotic stresses, and nutrient status [50,51]. The nutritional status of plants plays an important role in supporting plant growth, and various studies have demonstrated that supplementation of vermicompost provides nutrients to the plants in readily available forms such as nitrogen and enhances plant nutrient-uptake [52]. Thus, the production of the resulting phenolics and flavonoids are low when plants are not nutrient-limited.

The degradation of the compounds following extract storage that was observed in this study was consistent with previous findings [53], where the total phenolic content in Cornelian cherries extracts was found to decrease after being stored at 2 °C for up to 60 days. A drastic decrease was observed in the total phenolic and flavonoid content of *Anemopsis californica* extracts after being stored at 4 °C [54]. The decrease in total phenolics and flavonoids after prolonged storage was caused by various factors including oxidation by enzymes such as glycosidase, phenolase, and polyphenol oxidase (PPO), degradation of compounds, the polymerization of these compounds with proteins, and the conversion between free and bound phenolic substances [55,56,57]. PPO is an enzyme that causes the oxidation of phenolic compounds to quinones [58] by using polyphenols as the substrate [59]. Additionally, sample processing could cause disruption of the cell structure, which results in the loss of total phenolics and flavonoids [60]. Conversely, the decrease in total phenolic content may be due to non-enzymatic reactions that can contribute to the loss of total phenolic and flavonoid contents after storage. For example, the hydrogen peroxide produced as the by-product from the oxidation of ascorbic acid to dehydroascorbic acid can adversely affect the production of phenolic compounds [61], and the production of ascorbic acid and dehydroascorbic acid has been reported to increase due to chilling stress [62].

Results of the current study indicate that organic fertilization through supplementation with vermicompost contributed to the stability of the bioactive compounds during storage. At present, no published scientific literature was found on the exact mechanisms behind this observation. However, we postulate that the chemical compounds present in the plants supplemented with vermicompost (VC and MF) may undergo less oxidation, less degradation, and less polymerization compared with the compounds present in plants supplemented with chemical fertilizer (CF) and control plants (CC). Moreover, soil and fertilizer application has been found to affect protein composition and concentration in plants [63], which can interact with plant polyphenols. Various scientific literature has reported that the covalent interaction between plant polyphenols and proteins can further influence the content of free polyphenols and their antioxidant capacity during processing, transportation, and storage [64,65,66]. This implies that further studies involving multi-omics platforms, such as through metabolomics and proteomics, are essential for better understanding of the dynamics of protein and compound synthesis in relation to fertilization practices, as well as their interactions.

## 4. Conclusions

Vermicompost does not exert any significant effect on the plant antioxidant activity and the composition of bioactive compounds. However, the usage of vermicompost produced plants with the same content of bioactive compounds as the control and as the plants supplied with chemical fertilizer. Different storage conditions affected the stability of the bioactive compounds present in the methanolic extracts; however, plants supplemented with vermicompost were found to exhibit better stability when stored at different storage conditions.

## Figures and Tables

**Table 1:** Effect of plant growth supplements on availability of phytoconstituents in methanolic extracts of *Clinacanthus nutans* leaves.

Phytochemical	Treatment			
	No Fertilizer (CC)	NPK Fertilizer (CF)	Vermicompost (VC)	Mixed Fertilizer (MF)
Alkaloid	−	−	−	−
Tannin	−	−	−	−
Phenol	+	+	+	+
Flavonoid	+	+	+	+
Saponin	+	+	+	+

+ Present; − Absent.

**Table 2:** Effect of plant growth supplementation on total phenolic, anthocyanin, and flavonoid contents in methanolic extracts of *C. nutans* leaves.

Treatment	Sample ID	Total Anthocyanin Content (µg/g DW)	Total Phenolic Content (mg GAE/g DW)	Total Flavonoid Content (mg QE/g DW)
No fertilizer	CC	2180.14 ± 338.43 ^a^	120.48 ± 6.70 ^a^	276.25 ± 3.09 ^b^
NPK fertilizer	CF	1933.52 ± 66.06 ^a^	181.53 ± 35.58 ^b^	256.66 ± 45.43 ^b^
Vermicompost	VC	1742.86 ± 62.30 ^a^	98.06 ± 2.27 ^a^	170.42 ± 7.55 ^a^
Mixed fertilizer	MF	1669.91 ± 122.12 ^a^	97.47 ± 18.73 ^a^	157.30 ± 26.42 ^a^

Note: Means with different letters within the same column are significantly different at *p* ≤ 0.05 according to Duncan’s multiple range test (DMRT).

**Table 3:** Effect of plant growth supplements on antioxidant potential of methanolic extracts of *C. nutans* leaves.

Treatment	Sample ID	DPPH IC_50_ (mg/mL)	ABTS IC_50_ (mg/mL)
No fertilizer	CC	1.18 ± 0.05 ^a^	0.98 ± 0.05 ^a^
NPK fertilizer	CF	1.41 ± 0.02 ^b^	1.15 ± 0.16 ^a,b^
Vermicompost	VC	1.67 ± 0.04 ^c^	1.41 ± 0.01 ^b,c^
Mixed fertilizer	MF	2.23 ± 0.02 ^d^	1.67 ± 0.17 ^c^

Note: Means with different letters within the same column are significantly different at *p* ≤ 0.05 according to Duncan’s multiple range test (DMRT).

**Table 4:** Pearson’s correlation coefficients between the variables.

Variables	TFC	TPC	TAC	DPPH	ABTS
TFC	1				
TPC	0.834 **	1			
TAC	0.399 **	0.429 **	1		
DPPH	−0.465 **	−0.464 **	−0.518 **	1	
ABTS	−0.376 **	−0.401 **	−0.427 **	0.545 **	1

** Correlation is significant at *p* < 0.01.
